# The Different Immune Responses by Age Are due to the Ability of the Fetal Immune System to Secrete Primal Immunoglobulins Responding to Unexperienced Antigens

**DOI:** 10.7150/ijbs.67203

**Published:** 2022-01-01

**Authors:** Jangho Lee, Kyoungshik Cho, Hyejin Kook, Suman Kang, Yunsung Lee, Jiwon Lee

**Affiliations:** R&D Center of Stemmedicare Ltd, Seoul, 06095, Republic of Korea.

**Keywords:** natural antibody, IgG3, fetal immune system, extracellular vesicles, SARS-CoV-2

## Abstract

Among numerous studies on coronavirus 2019 (COVID-19), we noted that the infection and mortality rates of severe acute respiratory syndrome coronavirus 2 (SARS-CoV-2) increased with age and that fetuses known to be particularly susceptible to infection were better protected despite various mutations. Hence, we established the hypothesis that a new immune system exists that forms before birth and decreases with aging.

**Methods:** To prove this hypothesis, we established new *ex-vivo* culture conditions simulating the critical environmental factors of fetal stem cells (FSCs) in early pregnancy. Then, we analyzed the components from FSCs cultivated newly developed *ex-vivo* culture conditions and compared them from FSCs cultured in a normal condition.

**Results:** We demonstrated that immunoglobulin M (IgM), a natural antibody (NAb) produced only in early B-1 cells, immunoglobulins (Igs) including IgG3, which has a wide range of antigen-binding capacity and affinity, complement proteins, and antiviral proteins are induced in FSCs only cultured in newly developed *ex-vivo* culture conditions. Particularly we confirmed that their extracellular vesicles (EVs) contained NAbs, Igs, various complement proteins, and antiviral proteins, as well as human leukocyte antigen G (HLA-G), responsible for immune tolerance.

**Conclusion:** Our results suggest that FSCs in early pregnancy can form an independent immune system responding to unlearned antigens as a self-defense mechanism before establishing mature immune systems. Moreover, we propose the possibility of new solutions to cope with various infectious diseases based on the factors in NAbs-containing EVs, especially not causing unnecessary immune reaction due to HLA-G.

## Introduction

Coronavirus 2019 (COVID-19), which has generated worldwide pandemics, has caused previously unexperienced phenomena, such as the rapid expansion of viruses before activating the innate immunity 1, increased infection and mortality rates due to continuous mutations 2, 3, severe side-effects across the whole body including the respiratory tract and nervous system 4, induction of incomplete and delayed adaptive immune response 5, 6, re-infection after cure 7, 8, and post-vaccination breakthrough infection 9. Particularly, we paid attention to the patterns of the asymptomatic infection rate in the young and the relatively high fatality rate of COVID-19 in the elderly. The case fatality rate (CFR) of seasonal influenza was less than 0.1%, whereas the CFR of COVID-19 was 1.38%, but was reported to be much higher at 13.4% in those over 80 years of age 10. In contrast, the CFRs of 0-9 years old children and 10-19 years old adolescents were reported to be very low compared to adults, 0.0026% and 0.0148%, respectively 10. In addition, many studies 11-14 reported that children's relatively less experienced immune system could eliminate severe acute respiratory syndrome coronavirus 2 (SARS-CoV-2) much faster than adults'. In particular, fetuses and newborns considered to be susceptible to viral infection also show a relatively low infection rate 15, but the exact cause is not yet known.

However, from the studies that children infected with seasonal coronavirus already have antibodies (Abs) that cross-react to unexperienced SARS-CoV-2 16, that live vaccines vaccinated in newborns activate non-specific immunity that has a protective effect against a variety of antigens within 2-3 days, much faster than target pathogen-specific immunity, which is typically activated over several weeks in adults who need to get the flu vaccine every year 17, and that SARS-CoV-2-specific IgM with a macromolecular structure that cannot be transmitted from the mother infected with SARS-CoV-2 exists in infant blood 18, we have established a hypothesis that another immune system may exist even before birth capable of protecting the fetus before the existing innate and adaptive immune systems can respond to new antigens after birth.

To prove this, we focused on fetal stem cells (FSCs) in early pregnancy before the mature immune systems were formed by various immune cells. For the first time, we confirmed that various immunoglobulins (Igs) such as IgM, IgG, IgA, and IgE could be secreted from FSCs around 10 weeks gestation, before 12-13 weeks when B cells, known as the only cells secreting Igs, appear in fetal blood and spleen 19. In addition, unlike stem cells from other origins, we demonstrated that only FSCs cultured under established *ex-vivo* culture conditions simulating *in vivo* environment during pregnancy were induced to secrete IgM, a natural antibody (NAb) that binds to and removes various foreign antigens as soon as infection occurs 20, and IgG3 that has the broadest range of antigen-binding, variability capable of binding to various antigens, the highest activation capability of complement system after antigen binding, and binding affinity with various immune cells among other Igs 21. Furthermore, we confirmed that secretions of complement proteins contributing to the innate immune system with NAbs, including IgM and IgG3, and various anti-viral proteins inhibiting infection on cell membranes and inside cells, not Igs' activity area, were also induced from them. This study suggests the possibility of the existence of a new immune system, named “Primal Immune System” that can protect FSCs from unexperienced external infectious agents by themselves before forming innate and adaptive immune systems through the interaction of various types of immune cells, and propose the mechanism by which the severity of COVID-19 increases with aging by decreased adaptability to new antigens based on the primal immune system. Furthermore, through the considerations of maternal mechanism to protect the fetus, fetal self-protection mechanism, multilayer defense mechanism depending on the infection route, and the blocking mechanism to immune evasion of the virus, we ultimately present the possibility of new solutions to promptly respond to various infectious diseases and disturbances in the immune system without causing unnecessary immune rejection, based on multiple factors that constitute the primal immune system, especially immune-tolerized extracellular vesicles containing newly proposed “Primal Immunoglobulin” which can promptly respond to unexperienced antigens.

## Methods

### Cell Lines and Culture

Human trophoblast (TBC) BeWo cells and their culture medium were purchased from ATCC (Manassas, VA, USA). BeWo cells were cultured in F-12k medium (ATCC) supplemented with 100 U/mL of penicillin (Sigma-Aldrich, St. Louis, MO, USA), 100 μg/mL of streptomycin (Sigma-Aldrich), and 10% fetal bovine serum (FBS) (Gibco by Thermo Fisher Scientific, Waltham, MA, USA). Human fetal stem cells (FSCs) were established using a known method from amniotic fluid obtained from the healthy pregnant woman at 10 weeks of gestation who performed amniocentesis with written informed consent for utilization of tissues and all experimental procedures approved by the Institutional Bioethics Committee of Stemmedicare Ltd. (SIRB-2020-AUG-01). Human hematopoietic stem cells (HSCs) were purchased from ATCC and were maintained in Hank's Balanced Salt Solution (HBSS) medium (ATCC) supplemented with 100 U/mL of penicillin, 100 μg/mL of streptomycin, and 10% heat inactivated FBS. Human amniotic mesenchymal stem cells (hAM-MSCs) and human umbilical cord blood mesenchymal stem cells (UCB-MSCs) were purchased from ScienCell (Carlsbad, CA, USA). Human amniotic fluid-derived mesenchymal stem cells (hAF-MSCs) were established as described in the previous study 22. These MSCs were maintained in DMEM medium (Welgene, Republic of Korea) supplemented with 100 U/mL of penicillin, 100 μg/mL of streptomycin, and 10% heat-inactivated FBS.

### *In vivo*-like temperature change, pH, and circulation conditions

An *in vivo*-like culture condition for inducing immune-tolerized cell lines was applied using temperature change, pH, and circulation conditions established in the previous study 22. In summary, temperature change between 36.0 ℃ and 37.0 ℃ with a five or six-day cycle and an acidic pH of 6.2 to 6.8 by hyaluronic acid (HA)-based matrix were applied to induce the secretion of various pregnancy-related hormones as shown in **Figure 2C**. Simultaneously, a circulation condition with a 24-hr cycle, as shown in **Figure 2D**, was applied to facilitate the signal transduction between the culture matrix and immune-tolerized cells to promote autocrine of various soluble factors.

### Establishment of co-culture system of HSCs and UCB-MSCs

An indirect co-culture system of HSCs and UCB-MSCs was established using a multi-dish with polycarbonate membrane insert (Thermo Fisher Scientific). HSCs were seeded at a density of 2 × 10^5^ / cm^2^ in a 6-well plate, and UCB-MSCs were seeded at a density of 2 × 10^4^ / cm^2^ in a 0.4 μm pore insert (Nunc). They were cultured in DMEM serum-free medium in a 1% O2 and 5% CO_2_ humidified atmosphere at 37°C for 120 hrs to obtain the conditioned medium to isolate EVs later.

### EVs Preparation

#### Multi-stage Filtration

To increase the efficiency of the subsequent stage filtration and recovery a high yield of EVs, multi-stage filtration method was applied, established in the previous study 22. In summary, the cell culture supernatant, centrifuged at 1,500 RPM for 5 min, was filtered with a 0.8-μm filter to remove cell debris and apoptotic bodies completely, and the supernatant was filtered with a 0.45-μm filter twice. To obtain small EVs, the supernatant was filtered with a 0.22-μm filter in the same manner as above.

#### AF/AM-MSC^CO^-EVs

The conditioned medium obtained from serum-free co-cultivation of hAM-MSCs and hAF-MSCs in multi-dish plate, as described in the previous study 22, was centrifuged at 1,500 RPM for 5 min at the room temperature, and the supernatant was filtered with a 0.45-µm filter after multi-stage filtration to obtain AF/AM-MSC^CO^-EVs.

#### HSC/UCB-MSC^CO^-EVs

The conditioned medium obtained from serum-free co-cultivation of HSCs and UCB-MSCs in multi-dish plate was centrifuged at 1,500 rpm for 5 minutes at the room temperature, and the supernatant was filtered with a 0.22-μm filter after multi-stage filtration to obtain HSC/UCB-MSC^CO^-EVs.

#### itTBC-EVs

BeWo cells with both syncytiotrophoblast (STB) and extravillous trophoblast (EVT) phenotypes were sub-cultured in HA-Matrix 3-5 times to establish immune-tolerized trophoblast cells (itTBCs). The conditioned medium, obtained from serum-free cultivation of itTBCs with a seeding density of 2.0 × 10^4^/cm^2^ in HA-Matrix containing AF/AM-MSC^CO^-EVs with *in vivo*-like temperature change, pH, and circulation conditions, was centrifuged at 1,500 RPM for 5 minutes at the room temperature, and the supernatant was filtered with a 0.45-μm filter after multi-stage filtration to obtain itTBC-EVs.

#### NAbs-FSC-EVs

The conditioned medium, obtained from serum-free cultivation of NAb-secreting FSCs (NAbs-FSCs) with a seeding density of 2.0 × 10^4^ / cm^2^ alone without any matrix after washing with phosphate buffered saline (PBS) (Welgene) several times to remove any contaminant during the cultivation on HA-matrix containing itTBC-EVs and HSC/UCB-MSC^CO^-EVs with *in vivo*-like temperature change, pH, and circulation conditions, was centrifuged at 1,500 RPM for 5 minutes at the room temperature, and the supernatant was filtered with a 0.22-μm filter after multi-stage filtration to obtain NAbs-FSC-EVs.

### Nanoparticle Tracking Analysis (NTA) measurement of EVs with Nanosight NS300

The concentration and size of EVs were analyzed by NTA using a NanoSight NS300 with a Blue 488 nm laser (Malvern Panalytical, Malvern, UK). All samples were diluted in PBS to reach concentrations inside the precision range of the NTA machine (2 × 10^8^ to 10 × 10^8^ particles/ml). EVs were measured at camera level 14 (camera shutter speed: 21.48 shutters/ms, slider gain: 366). After capture, the videos have been analyzed using the in-build NanoSight Software NTA 3.4 Build 3.4.003 with a detection threshold 5.

### Flow Cytometry

The expressions of Igs, NAbs, stem cell and EV-specific markers, and HLA-G proteins in FSCs and FSCs-derived EVs were quantified by flow cytometry. FSCs were harvested and stained with anti-Human CD73 APC (Invitrogen by Thermo Fisher Scientific, Waltham, MA, USA), anti-Human CD90 APC (Invitrogen), anti-Human CD105 APC (Invitrogen), anti-Human SSEA-4 AF488 (Invitrogen), anti-HLA-G 87G APC (BioLegend, San Diego, CA, USA), anti-HLA-G 2A12 FITC (Invitrogen), anti-Human IgG Fc AF488 (BioLegend), anti-Human IgG3 Hinge AF555 (SouthernBiotech, Birmingham, AL, USA), anti-Human IgG3 AF488 (Novus Biologicals, Centennial, CO, USA), and anti-Human IgM AF647 (BioLegend) Abs for 30 min at room temperature. Intracellular staining of FSCs were performed using Intracellular Fixation & Permeabilization Buffer set (Invitrogen) according to the manufacturer's instruction.

The EVs were stained with anti-Human CD9 FITC (Invitrogen), anti-Human CD63 PE (Invitrogen), and CD81 APC (Invitrogen) Abs for 30 min at room temperature. Intracellular staining of EVs were performed after EV isolation using Exosome Isolation and RNA Purification Kit (System Biosciences, Palo Alto, CA, USA) according to the manufacturer's instruction. Isolated EVs were treated with permeabilizing solution for 1 hour at room temperature and stained with anti-HLA-G 2A12 FITC, anti-Human IgG Fc AF488, anti-Human IgG3 AF488, and anti-Human IgM AF647 Abs for 30 min at room temperature. Then, stained EVs were isolated again using Exosome Isolation and RNA Purification Kit and analyzed through flow cytometry using various sizes of reference beads (Thermo Fisher Scientific).

The flow cytometry analysis of FSCs and their EVs were performed using Novocyte Quanteon (Agilent Technologies, Santa Clara, CA, USA), and the data were analyzed by Novoexpress software ver. 1.43.

### Enzyme-linked immunosorbent assay (ELISA)

The concentration of sHLA-G (shedding HLA-G1 and HLA-G5) was detected by Human HLA-G ELISA Kit (LSBio, Seattle, WA, USA) using MEM-G/9 Ab according to the manufacturer's instruction. The concentration of soluble HLA-G isoforms (HLA-G5 and HLA-G6) was detected by soluble HLA-G ELISA Kit (MyBioSource, San Diego, CA, USA) using 5A6G7 Ab according to the manufacturer's instruction.

The concentrations of IgG3 and IgM were analyzed using Human IgG3 ELISA Kit (MyBioSource) and Human IgM ELISA Kit (Abnova, Taipei, Taiwan) according to the manufacturer's instructions, respectively.

### Protein Antibody Array (PAA)

The Ig and protein profiles in EVs secreted from FSCs were analyzed using SET100 Signaling Explorer Antibody Array (Full Moon Biosystems, Sunnyvale, CA, USA) and Human L493 Array (RayBiotech, Peachtree Corners, GA, USA) according to the manufacturer's instructions.

### Cytokine Array Analysis of EVs

The concentrations of human growths and cytokines in EVs were analyzed by Quantibody Human Cytokine Array 1000 (RayBiotech) according to the manufacturer's instruction.

### Protein absolute quantification of EVs

ESI-Q-TOF MS/MS analysis is performed on pre-treated EV sample according to the manufacturer's instructions using Synapt G2-Si HDMS (Waters, Milford, MA, USA) equipment. Protein identification was performed using the human database (version 3.87) of the IPI (International Protein Index). Protein absolute quantification was carried out based on the standard BSA mass value information (SwissProt) according to the manufacturer's instructions and the method suggested by Silva J. et al. 23.

### Characterization of EVs with ExoView^TM^ R100

Co-expressions of CD9, CD61, CD81, syntenin, IgG3, and IgM in EVs were analyzed using Tetraspanin Custom Kit with ExoView^TM^ R100 (NanoView Biosciences, Boston, MA, USA) according to the manufacture's instruction of Cargo and Surface Membrane Immuno-Fluorescence Staining. Labelling Abs that consist of anti-syntenin Alexa-555, anti-human IgG3 Alexa Fluor 488 (Novus Biologicals), and anti-human IgM Alexa Fluor 647 Abs (BioLegend), were used. The data were then analyzed using ExoViewer Analyzer 3.0 with sizing thresholds set to 50 to 200 nm diameter.

### Statistical analysis

Statistical analyses were performed using the Student's *t*-test for the comparison of means in more than two groups. Data presented are mean±S.D. and *P*<0.05 was considered statistically significant.

## Results

**Figure 1:**
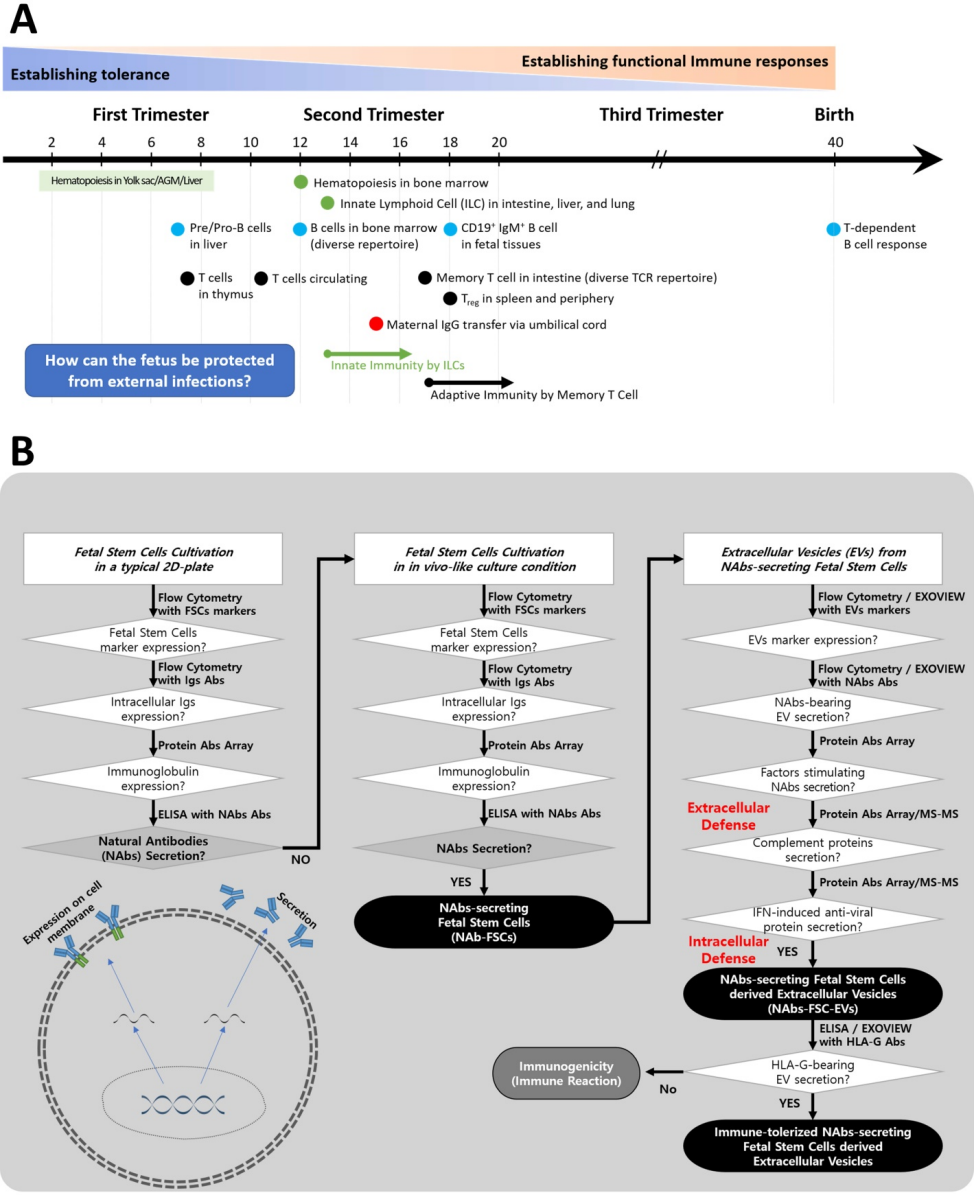
** A schematic diagram of the process of verifying hypotheses to identify a new immune system built up by FSCs in early pregnancy. (A)** The developing progress of the fetal immune system. Igs-secreting B cell progenitors and pre pro-B cells were detected only in the fetal liver around 9 weeks of gestation, and mature B cells expressing B cell receptors were found in various fetal tissues after 18 weeks of gestation. In addition, maternal IgG can be transferred to the fetus through the umbilical cord after 15 weeks of gestation. However, it is not known whether the fetus produces Igs before the innate and adaptive immune system are formed. Adapted from 25 with permission from AAAS. **(B)** To investigate the mechanism of self-protection by FSCs before establishing the innate and adaptive immune system by immune cells in early pregnancy, we established hypotheses and prepared a flow chart to verify them. (1) We confirmed whether Igs, particularly NAbs, including IgG3, which act on the innate immune system, were secreted from FSCs under a typical 2D culture condition. (2) We established an *ex-vivo* culture condition that simulates *in vivo* environment of FSCs to induce the secretion of NAbs from them. We confirmed the secretions of Igs and NAbs from FSCs^Ex-vivo^ (NAbs-FSCs) cultured in this culture condition and compared them with FSCs^Control^ cultured in a typical 2D culture condition. (3) We confirmed NAbs, proteins related to the promotion of Ig secretion, and complement proteins in NAbs-FSC-EVs secreted from NAbs-FSCs cultured in a newly established *ex-vivo* culture condition to induce the secretion of NAbs and compared them with FSCs^Control^-EVs. In addition, we confirmed the difference in induction aspects according to the culture conditions by comparing the content of HLA-G proteins inducing immune tolerance and various anti-viral proteins induced by IFN in NAbs-FSC-EVs with FSCs^Control^-EVs. Moreover, we compared and analyzed the induction aspects of NAbs such as IgG3 and IgM in NAbs-FSC-EVs and FSCs^Control^-EVs through Exoview®. This verification process confirmed that FSCs cultivated in a newly established *ex-vivo* culture condition could secrete NAbs, complement proteins, and various anti-viral proteins, which could construct the new immune system to protect themselves from external infectious agents at the cellular level.

Igs secreted by B lymphocytes through somatic hypermutation (SHM) and class-switch recombination (CSR) are found in fetal tissues and serum after 20 weeks of gestation 24, and the period of formation of Abs through T cell-dependent B cell responses is known after birth 25. However, it is known that IgG, the only Ig that can pass through the placenta, is delivered through the umbilical cord formed around 15 weeks of pregnancy to protect the fetus from various sources of infection 26. Still, it is not precisely known whether Igs were formed in fetuses before that time (**Figure 1A**).

Therefore, we established the flowchart as shown in **Figure 1B** to confirm the possibility of secretion of Igs from FSCs in early pregnancy and to clarify the concept of a new immune system built up by FSCs by developing an *ex-vivo* culture system that can induce the secretion of various factors, including Igs, which can protect themselves from foreign infections.

### Confirming the possibility that FSCs in early pregnancy can produce Igs

There have been reports that FSCs expressed the characteristics of HSCs 27-29. However, no study has reported that FSCs could secrete Igs produced only from B cells of HSC lineage. To confirm the possibility of Igs production in the fetus in the early pregnancy, we obtained FSCs from amniotic fluid samples donated from a healthy pregnant woman at around 10 weeks of gestation for amniocentesis. Through flow cytometry analysis on the obtained FSCs, we confirmed the expressions of various stem cell markers and Igs. Then, we performed PAA and ELISA analysis on the conditioned media obtained by culturing FSCs on a typical 2D plate. As a result, we confirmed that IgM, IgG, IgA, and IgE were expressed at very low levels in microarray analysis. Still, no Ig, especially NAbs acting on the innate immunity, was detected in ELISA analysis. From these results, we confirmed the possibility of Ig expression in FSCs. However, it was confirmed that establishing a new culture condition simulating *in vivo* environment capable of inducing NAbs secretion from FSCs is necessary to verify the possibility of secretion of NAbs.

### Establishment of an *ex-vivo* culture condition simulating *in vivo* environment during pregnancy to induce the secretion of NAbs in FSCs

In a previous study 22, we established the maternal-fetal interface-like *ex-vivo* culture condition to investigate the immune tolerance mechanism that completely protects the fetus from the maternal immune system during pregnancy. In addition, we demonstrated for the first time that EVTs cultured in this condition promoted the continuous expression and secretion of HLA-G, which induces an immune tolerance environment through the activation of self-secretion of pregnancy-related hormones, including human chorionic gonadotropin (hCG) and progesterone (PR). In particular, we confirmed that the secretion of hCG and PR from TBCs could be promoted cultured in maternal-fetal interface-like *ex-vivo* culture condition by applying temperature profile based on the woman's body temperature change, pH, and vibration conditions to replace the internal nervous system that regulates the secretion of hormones promoting the expression and secretion of HLA-G protein that protects the fetus from the mother's immune cells. As a result, we could establish immune-tolerized trophoblasts (itTBCs) that express and secrete HLA-G consistently. Based on this, we tried to establish new *ex-vivo* culture conditions to induce the secretion of NAbs from FSCs in early pregnancy.

**Figure 2:**
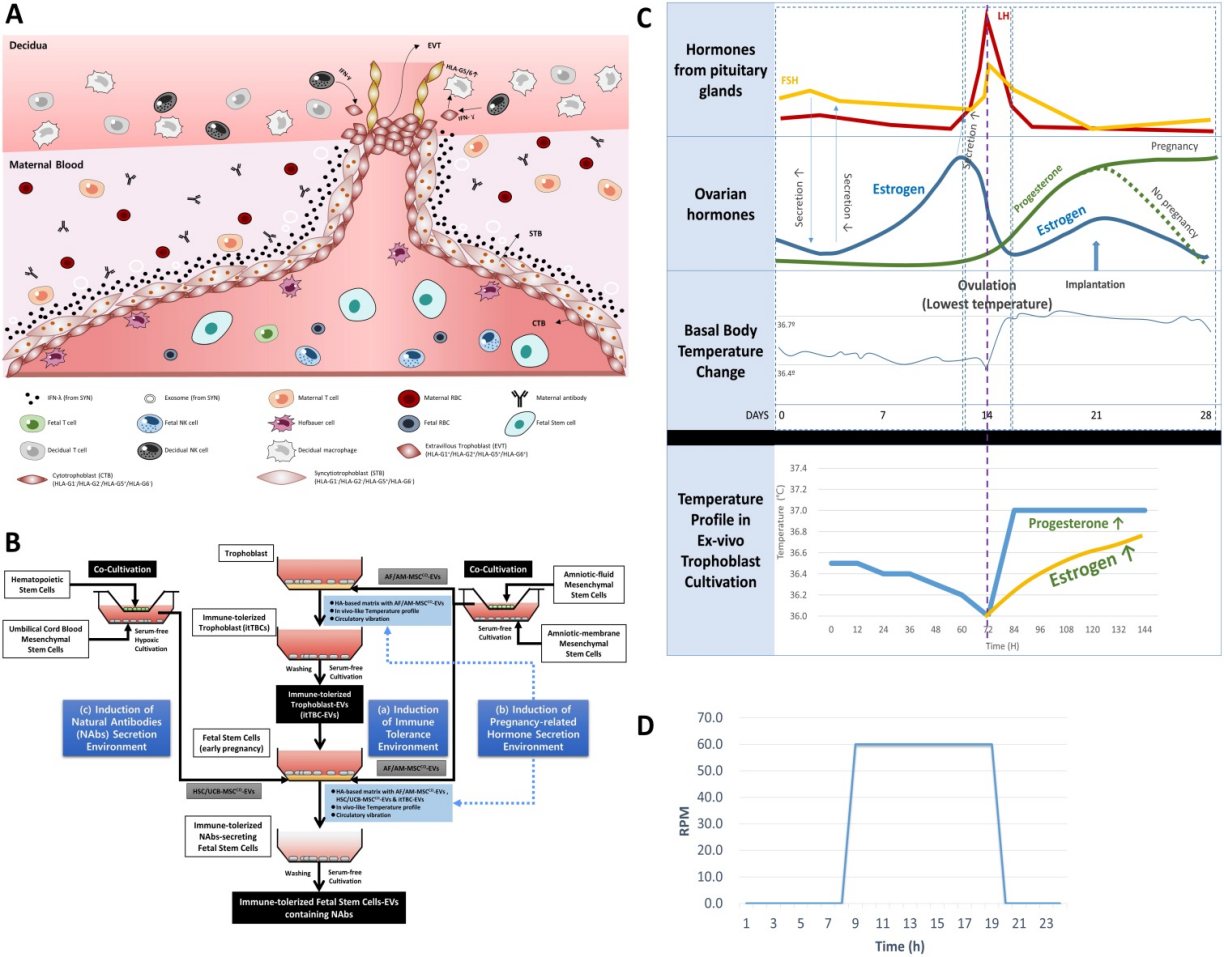
** Establishment of new *ex-vivo* culture conditions to induce the secretion of NAbs in FSCs in early pregnancy. (A)** *In vivo* environment of FSCs in the first trimester of pregnancy and factors inducing the secretion of NAbs in FSCs. **(B)** A schematic diagram of novel *in vivo* environment-like *ex-vivo* culture conditions inducing NAbs secretion in FSCs. **(C)** An *ex-vivo* cell culture temperature profile with a 5-6 day-cycle simulating the 28-day cycle of female hormones and body temperature changes applied to new *ex-vivo* culture conditions to induce hormone secretion in TBCs and FSCs replacing the internal nervous system 22. **(D)** A graph of 24-hour-cycle vibration condition applied to new *ex-vivo* culture conditions for inducing pregnancy-related hormone secretion in TBCs and FSCs 22.

FSCs in the first trimester of pregnancy are protected by layers of TBCs in direct contact with the maternal blood and decidua. Moreover, they coexist with immature immune cells of the fetus (**Figure 2A**). To simulate this *in vivo* environment of FSCs in the early stages of pregnancy, we devised three *in vitro* culture environment modules as shown in **Figure 2B**. The first is the maternal-fetal interface-like *in vitro* culture condition established in a previous study 22, in which we cultured TBCs on HA-based matrix for *in vitro* culture containing AF/AM-MSC^CO^-EVs obtained through co-culture of amniotic fluid and amnion membrane-derived stem cells for pH and physical conditions similar to *in vivo* environment during pregnancy. The second is an *in vitro* culture condition for inducing the secretion of pregnancy-related hormones. From the possibility of Ig production of FSCs that we have identified, we hypothesized that pregnancy-related hormones secreted from TBCs could induce HLA-G secretion protecting the fetus from the maternal immune system, and at the same time, affect the secretion of Igs, especially NAbs, protecting the fetus from foreign antigens. To prove this, we obtained itTBC-derived EVs (itTBC-EVs) containing various pregnancy-related hormones by applying the temperature profile and vibration conditions similar to women's internal environment to maternal-fetal interface-like culture conditions, as shown in **Figure 2C-D**. Lastly, to stimulate the secretion of NAbs from FSCs, we obtained HSC/UCB-MSC^CO^-EVs from serum-free co-cultivation of HSCs and UCB-MSCs under hypoxic conditions by replacing undeveloped fetal immune cells. And by applying these EVs together with itTBC-EVs, we prepared a new matrix for *in vitro* culture. Moreover, by applying the above temperature change, pH, and vibration conditions, we finally established the new *ex-vivo* culture condition simulating the *in vivo* environment to confirm the induction of NAbs secretion in FSCs.

### Establishment and characterization of FSCs secreting NAbs

**Figure 3:**
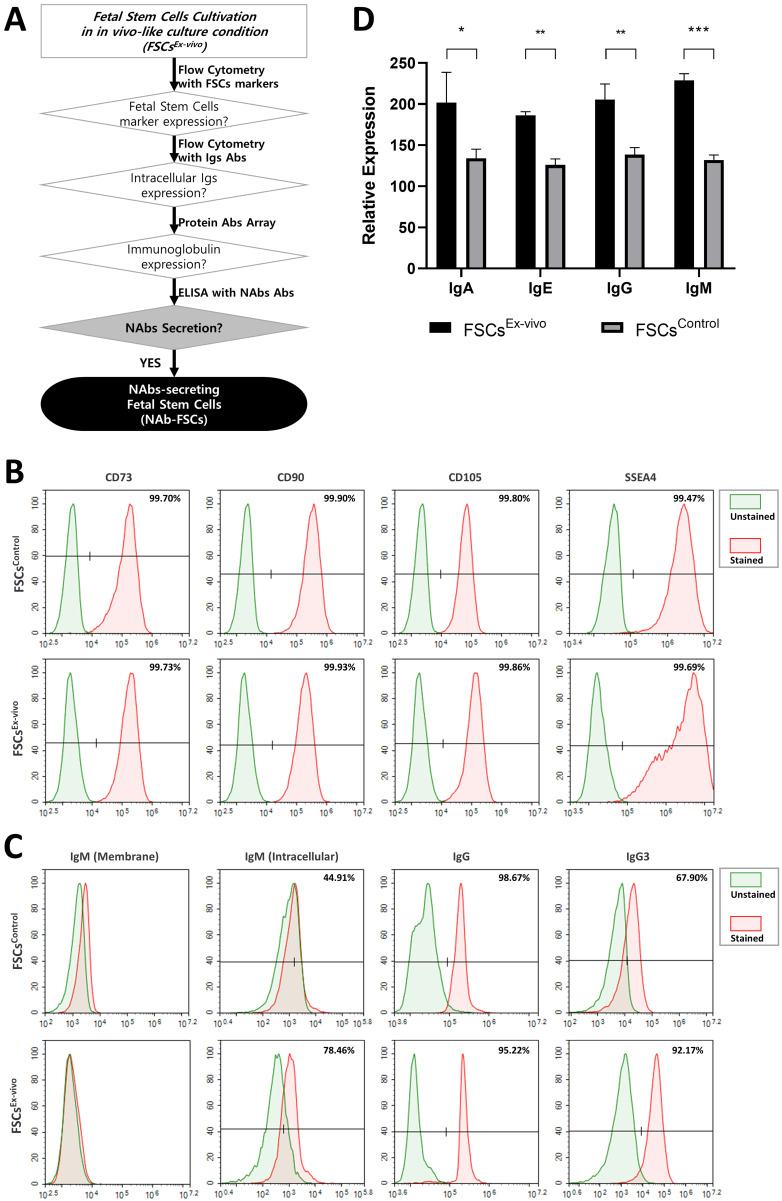
** Analysis of NAbs expression and secretion characteristics of FSCs cultured in the novel *ex-vivo* culture conditions for inducing the secretion of Nabs. (A)** A schematic diagram to verify NAbs expression and secretion potential in FSCs (FSCs^Ex-vivo^) cultured under new *ex-vivo* culture conditions. **(B)** Flow cytometry results comparing the expression patterns of stem cell-specific markers in FSCs cultured in FSCs^Control^ and FSCs^Ex-vivo^. The results showed that the expression aspects of MSC markers (CD73, CD90, and CD105) and ESC marker (SSEA4) were very similar in FSCs^Control^ and FSCs^Ex-vivo^. **(C)** Flow cytometry results comparing the cell membrane and intracellular expression patterns of Igs and NAbs in FSCs^Control^ and FSCs^Ex-vivo^. The expression of membrane-bound IgM is rather higher in FSCs^Control^ than FSCs^Ex-vivo^, but the expressions of intracellular IgM and IgG3 (NAbs) are much higher in FSCs^Ex-vivo^ than FSCs^Control^. The expression pattern of IgG is similar in FSCs^Control^ and FSCs^Ex-vivo^. **(D)** Results of protein antibody microarray analysis comparing the relative expression levels of Igs in the culture supernatants of FSCs^Control^ and FSCs^Ex-vivo^. Compared to FSCs^Control^, FSCs^Ex-vivo^ secreted higher levels of Igs. Error bars represent standard deviation. *, P <0.05; **, P<0.01; ***, P<0.001; Student's t-test.

The expression and secretion of NAbs in FSCs (FSCs^Ex-vivo^) cultured in new *ex-vivo* culture conditions established for inducing NAbs secretion were compared with those of FSCs (FSCs^Control^) cultured in general 2D-plate and verified. **Figure 3A** is a schematic diagram proving the expression and secretion of NAbs in FSCs^Ex-vivo^ through various analysis methods. **Figure 3B** shows the comparison of the expression patterns of stem cell-specific markers for FSCs^Control^ and FSCs^Ex-vivo^ through flow cytometry. As a result of performing flow cytometry using MSC markers (CD73, CD90, and CD105) and embryonic stem cell (ESC) markers (SSEA4) to investigate the cellular characteristics of FSCs in early pregnancy, which are known to have both ESC and MSC characteristics 27, it was confirmed that FSCs^Control^ and FSCs^Ex-vivo^ showed almost the same expression patterns. These results indicate that FSCs^Ex-vivo^ cultured in new *ex-vivo* culture conditions established by us fully maintain the inherent MSC and ESC characteristics. **Figure 3C** shows the flow cytometry analysis of the expression patterns of IgG and NAbs (IgM and IgG3) for FSCs^Control^ and FSCs^Ex-vivo^. As a result of analyzing the cell membrane expression of IgM, the only membrane-bound Ig, interestingly, some membrane-bound IgM was expressed in FSCs^Control^, but few expressed in FSCs^Ex-vivo^. However, as a result of analyzing intracellular expressions, the expression pattern of IgG was similar in FSCs^Control^ and FSCs^Ex-vivo^, but NAbs were expressed higher in FSCs^Ex-vivo^ than FSCs^Control^. These results confirmed that newly established *ex-vivo* culture conditions could induce the increases in intracellular expression of IgG3 and IgM in fetal stem cells.

**Figure 3D** shows the analysis of the Ig secretion patterns of FSCs^Control^ and FSCs^Ex-vivo^ through Protein Antibody Microarray. As a result of analyzing the expression pattern of Igs in the culture supernatant obtained through the serum-free culture of each FSC, we confirmed that the expression of all Igs such as IgG, IgM, IgE, and IgA included in the microarray were higher in FSCs^Ex-vivo^ than in FSCs^Control^. These results indicate that our *ex-vivo* culture conditions induced the expression and secretion of Igs in FSCs.

Finally, we verified the secretion of NAbs in FSCs^Ex-vivo^ through IgM and IgG3 ELISA analysis. To prove whether the expression and secretion characteristics of Igs in stem cells, not immune cells, are inherent to FSCs and whether new *ex-vivo* culture conditions induce the secretion of NAbs, IgM and IgG3 concentrations were analyzed by ELISA for each conditioned medium obtained by serum-free cultivation of various stem cell lines under normal 2D-plate culture conditions (2D) and new *ex-vivo* culture conditions (Ex-vivo). As shown in **Table 1**, IgG3 and IgM were not detected in the conditioned medium of FSCs cultured under normal culture conditions (2D), but only in the conditioned medium of FSCs cultured under newly established *ex-vivo* culture conditions (Ex-vivo). In addition, IgG3 and IgM were not detected in the conditioned medium of cord blood, bone marrow, adipose, and umbilical cord-derived MSCs and HSCs regardless of the culture conditions. Moreover, we confirmed that IgG3 and IgM were not detected in all EVs used in new *ex-vivo* culture conditions, including itTBC-EVs, AF/AM-MSC^CO^-EVs, and HSC/UCB-MSC^CO^-EVs. These results verified that only FSCs in the early stages of pregnancy have the characteristic of secreting NAbs to protect themselves from external infections, and FSCs^Ex-vivo^ cultured in established *ex-vivo* culture conditions are NAb-secreting FSCs (NAb-FSCs).

Meanwhile, TBCs secrete estrogen (ER), another critical sex hormone, together with PR and hCG during pregnancy 30. We found that hCG, PR, and ER were induced in itTBC-EVs used in new *ex-vivo* culture conditions. A study 31 showed that ER promotes Ig production in human peripheral blood mononuclear cells (PBMCs), but no study has been reported in other types of cells. Therefore, our results that Igs were induced in FSCs^Ex-vivo^ cultured in *ex-vivo* culture conditions applying itTBC-EVs containing ER at a higher level than in FSCs^Control^ obtained from cultivation without itTBC-EVs verify that ER also contributes to the promotion of Ig transcription in FSCs before B cell differentiation. Besides, our results demonstrate our hypothesis that hormones secreted during pregnancy can induce the secretion of HLA-G and Igs, thereby simultaneously protecting the fetus from the maternal immune system and foreign antigens.

### Characterization of NAb-secreting FSCs-derived EVs (NAbs-FSC-EVs)

**Figure 4:**
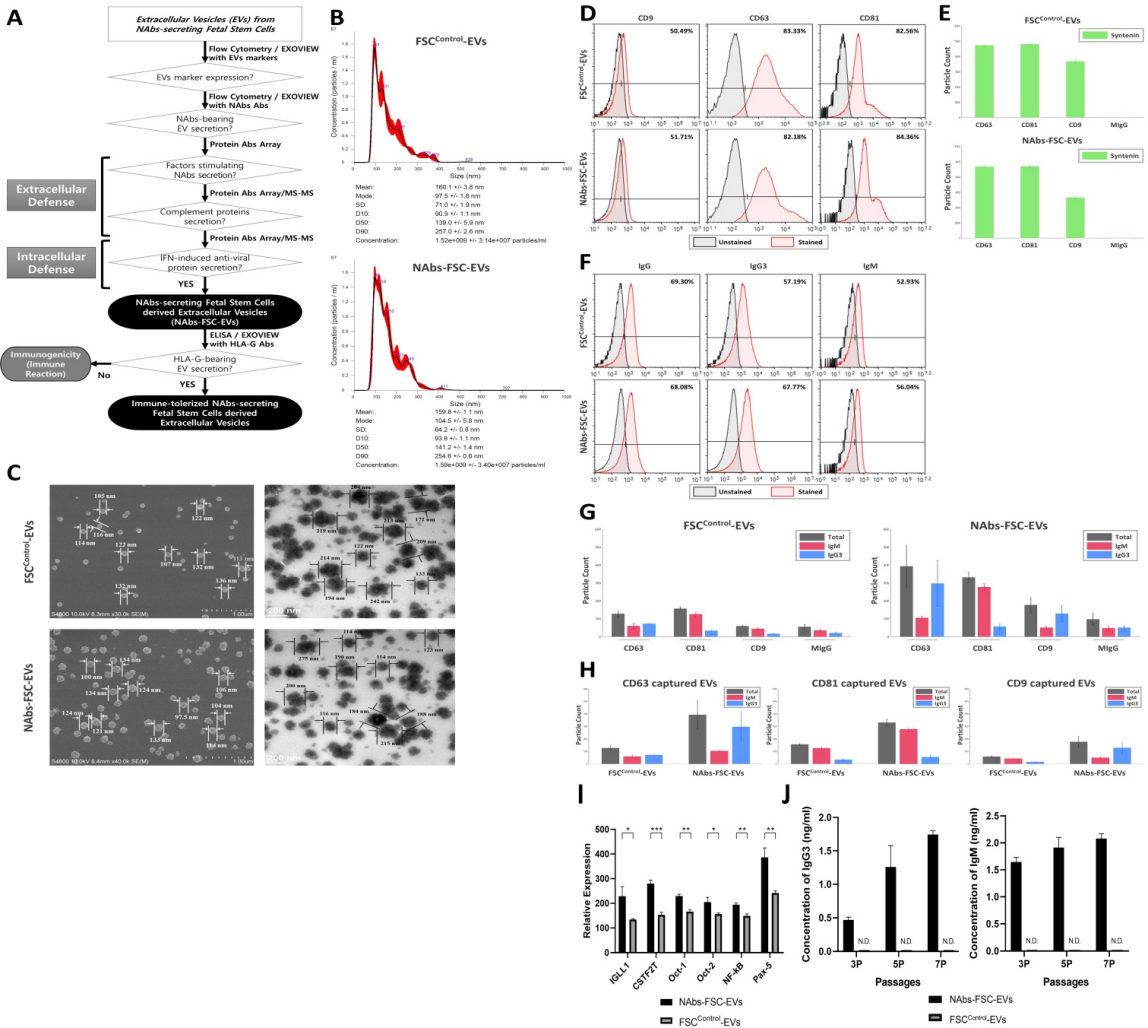
** Characterization of NAb-secreting FSCs-derived EVs (NAbs-FSC-EVs). (A)** A schematic diagram verifying whether NAbs are contained in NAbs-FSC-EVs secreted from NAbs-FSCs cultured under new *ex-vivo* culture conditions. **(B)** The results of NTA on NAbs-FSC-EVs and FSC^Control^-EVs secreted from FSCs^Control^ cultured on a typical 2D plate. The concentrations, average sizes, and distribution of FSC^Control^-EVs and NAbs-FSC-EVs are almost similar. There is no significant difference in EV secretion characteristics according to the culture conditions. **(C)** SEM (left) and TEM (right) images of FSC^Control^-EVs and NAbs-FSC-EVs, respectively. There are no significant differences in shapes and size distributions in both EVs according to the culture conditions. **(D)** The results of flow cytometry analysis to compare the expression patterns of exosome markers (CD9, CD63, and CD81) in FSC^Control^-EVs and NAbs-FSC-EVs. The expression aspects that CD63 and CD81 are expressed at higher levels than CD9 are similar in both EVs. **(E)** The results of co-expression analysis of exosome markers (CD9, CD63, CD81, and syntenin) in FSC^Control^-EVs and NAbs-FSC-EVs using ExoView®. They show expression patterns very similar to the flow cytometry results in Figure 4D, and these common expression patterns appear to be a unique characteristic of FSCs-derived EVs. **(F)** The results of flow cytometry analysis comparing the presence of IgG and NAbs (IgG3 and IgM) in FSC^Control^-EVs and NAbs-FSC-EVs. **(G)** The results of exosomal cargo analysis to compare the content of NAbs in FSC^Control^-EVs and NAbs-FSC-EVs using ExoView®. Similar to the flow cytometry results in Figure 4F, they show that NAbs-FSC-EVs contain higher levels of NAbs than FSC^Control^-EVs. **(H)** The results of analyzing the patterns of NAbs content in both EVs captured by each exosomal marker using Exoview®. **(I)** The results of protein antibody microarray analysis comparing relative expression levels of B cell-specific proteins related to the promotion of Ig transcription induced in FSC^Control^-EVs and NAbs-FSC-EVs. They show that B cell-specific proteins related to the promotion of Ig transcription are induced in NAbs-FSC-EVs at higher levels than FSC^Control^-EVs. **(J)** The results of analyzing the concentrations of IgG3 and IgM contained in both EVs obtained from the subculture of FSCs^Control^ and NAbs-FSCs using each ELISA kit. IgG3 and IgM were not detected in all FSC^Control^-EVs, but the contents of IgG3 and IgM in NAbs-FSC-EVs gradually increased as the subculture progressed. Error bars represent standard deviation. *, P <0.05; **, P<0.01; ***, P<0.001; Student's t-test; N.D., Not Detected.

To verify whether NAbs-FSC-EVs secreted from NAbs-FSCs contained NAbs, we performed various characterization and compared the results with FSCs^Control^-EVs secreted from FSCs^Control^. **Figure 4A** shows a schematic diagram demonstrating whether NAbs-FSC-EVs contain various factors, including NAbs, that can protect FSCs from external infection. **Figure 4B** shows the results of NTA to determine the size distribution and concentration of FSC^Control^-EVs and NAbs-FSC-EVs isolated by multi-stage filtration from the serum-free culture supernatant of 1.0 x 10^5^ FSCs at passage 2, respectively (n=3). As a result of NTA, we confirmed that the concentration, average size, and distribution of FSC^Control^-EVs and NAbs-FSC-EVs showed almost similar patterns. **Figure 4C** shows scanning electron microscope (SEM, left) and transmission electron microscope (TEM, right) images of FSC^Control^-EVs and NAbs-FSC-EVs, respectively, and we confirmed that they show very similar size distributions and shapes.

**Figure 4D** represents the results of flow cytometry analysis of the expression patterns of exosome (small EVs) markers, such as CD9, CD63, and CD81, in FSC^Control^-EVs and NAbs-FSC-EVs. Compared to CD63 and CD81, which show similar expression levels in both EVs, we confirmed that the patterns showing a somewhat low expression level of CD9 were similar in both EVs. This result seems to be due to the unique characteristics of FSCs. In addition, **Figure 4E** shows the results of re-verifying the co-expression patterns of exosome markers such as CD9, CD63, CD81, and syntenin in each EV using ExoView®, which is very similar to the flow cytometry results shown in **Figure 4D**. These results show no difference in the expression characteristics of the exosome markers of EVs secreted from each FSCs regardless of the culture conditions and also show that EVs used in the analysis are all normal exosomes.

**Figure 4F** show the comparison of IgG and NAbs (IgG3 and IgM) content in FSC^Control^-EVs and NAbs-FSC-EVs through flow cytometry. Similar to the results shown in **Figure 3C**, we confirmed that the levels of IgG content were similar in FSC^Control^-EVs and NAbs-FSC-EVs, but IgM and IgG3 were contained at slightly higher levels in NAbs-FSC-EVs than in FSC^Control^-EVs. In addition, **Figure 4G-H** show the results of exosomal cargo analysis using Exoview® to determine whether each EV contains NAbs. We again verified the flow cytometry analysis results (**Figure 4F**) that IgM and IgG3 were contained at a relatively higher level in NAbs-FSC-EVs than FSC^Control^-EVs. These results confirmed that the newly established *ex-vivo* culture conditions could induce the secretion of EVs containing NAbs from FSCs in early pregnancy.

Meanwhile, to study the mechanism by which NAbs are induced at higher levels in NAbs-FSC-EVs, we performed protein antibody array analysis on the components included in FSC^Control^-EVs and NAbs-FSC-EVs and compared the expression patterns of B cells specific proteins related to the transcription of Igs in both EVs (**Figure 4I**).

B cell-specific Ig gene expression and secretion are due to tissue-specific expression of octamer transcription factors (OCT), nuclear proteins that bind to the octamer sequence ATGCAAAT motif in the promoter and enhancer of the Ig heavy chain gene 32, and another transcription factor, nuclear factor kappa-light-chain-enhancer of activated B cells (NF-kB), is known as an enhancer of the Ig light chain gene 33. In addition, genes specially expressed in early B cells, immunoglobulin lambda-like polypeptide 1 (Igll1, also λ5) and paired box protein 5 (Pax-5), are also known to be expressed during B cell receptor (BCR) development which is converted into a membrane-bound form of IgM by antigen stimulation 34, 35. Furthermore, the cleavage stimulating factor (CSTF), which is known to be involved in the growth and differentiation of B cells, has been reported to convert the membrane-bound form of IgM mRNA to secreted form 36.

Our results shown in **Figure 4I** confirmed that various B cell-specific and Ig transcription-related proteins were expressed in FSCs at the stage before B cell differentiation and showed that these proteins are induced at higher levels in NAbs-FSC-EVs than FSC^Control^-EVs. In particular, our results that NAbs-FSC-EVs contained higher levels of soluble IgM than FSC^Control^-EVs while the expression of membrane-bound IgM was decreased in NAbs-FSCs than in FSCs^Control^ were due to the effect of CSTF induced at higher levels in NAbs-FSC-EVs. Our results represented that NAbs-FSCs established by environmental factors simulated to induce secretion of NAbs may have the ability to secrete higher levels of IgG3 and IgM.

**Figure 4J** shows the results of analyzing the contents of IgG3 and IgM in FSC^Control^-EVs and NAbs-FSC-EVs obtained by subculturing FSCs^Control^ and NAbs-FSCs in each culture condition by ELISA. As shown in [**Table 1**], both IgG3 and IgM were not detected in all FSC^Control^-EVs. But we confirmed that the concentration of IgG3 and IgM increased in NAbs-FSC-EVs as the subculture progressed. These results indicate that the properties of FSCs capable of secreting NAb-containing EVs are reduced under normal culture conditions, whereas their properties can be enhanced under newly established ex-vivo culture conditions to induce NAbs secretion.

### Self-defense mechanism by NAbs and complement proteins induced in NAbs-FSC-EVs

Innate immunity is a primary defense system that removes the foreign antigens by non-specifically immediately reacting to them and activates antigen-specific adaptive immunity. Various immune cells, including macrophages, dendritic cells (DCs), and natural killer (NK) cells, are involved in innate immunity, but their cytotoxicities are mainly induced by NAbs and complement proteins together that can bind to various antigens. Therefore, we investigated whether NAbs-FSCs secrete complement proteins capable of eliminating foreign antigens by binding with NAbs.

**Figure 5:**
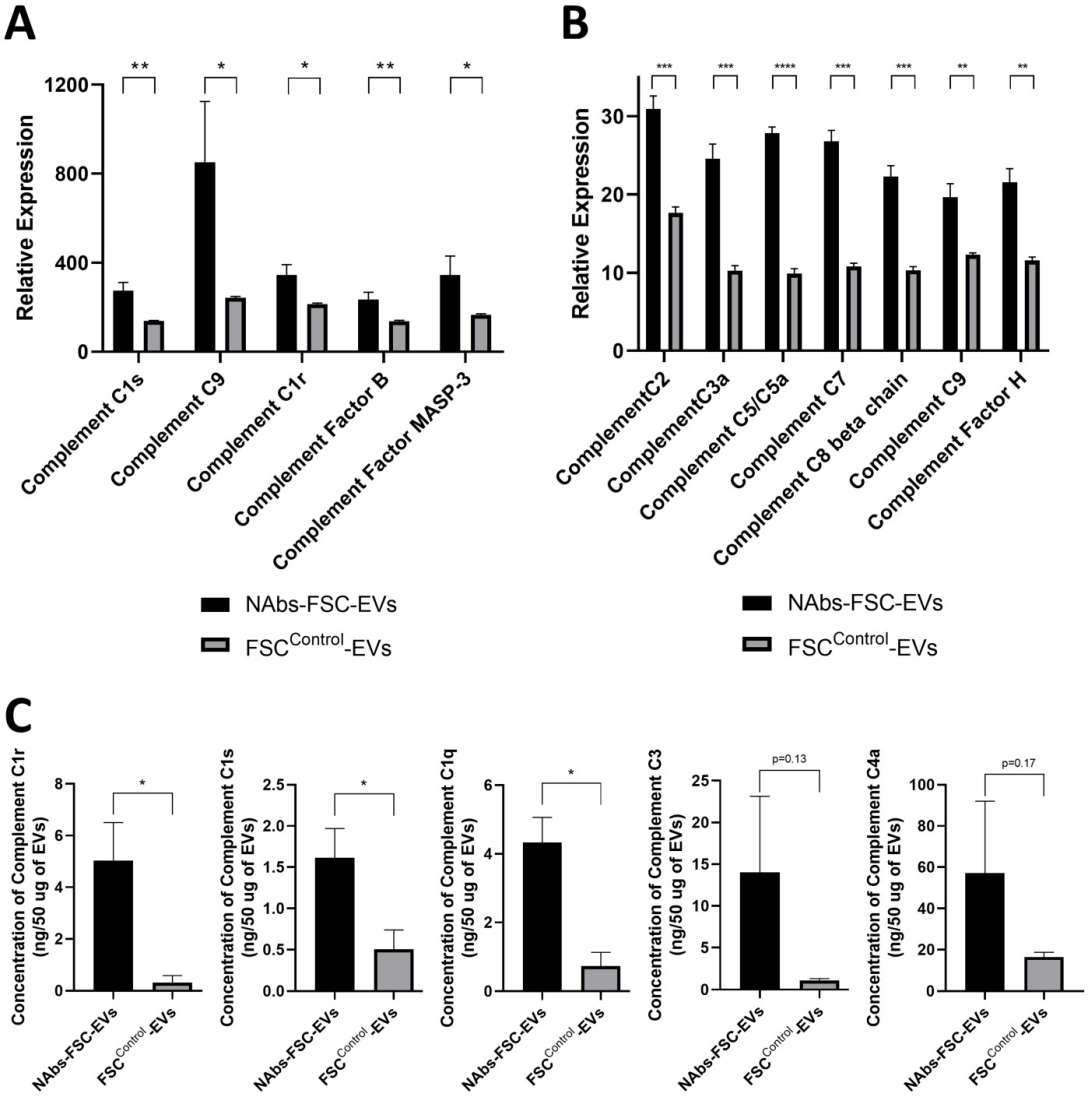
** Analysis results of various complement proteins contained in NAbs-FSC-Evs.** The complement system is activated by the classical pathway (CP), the alternative pathway (AP), and the lectin pathway (LP). C1 subcomponents such as C1q, C1r, and C1s form the C1 complex in the CP, and the C5, C6, C7, C8, and C9 complement proteins form the membrane attack complex. C3a, C4a, and C5a proteins, generated in this process, recruit various immune cells to spread the inflammatory response 37. Comparisons of relative expression levels of complement proteins contained in NAbs-FSC-EVs and FSC^Control^-EVs using SET100 protein antibody microarray **(A)** and human L493 array **(B)**. **(C)** Comparisons of absolute quantification of complement proteins contained in NAbs-FSC-EVs and FSC^Control^-EVs using ESI-Q-TOF MS/MS. We confirm that NAbs-FSC-EVs contain higher levels of various complement proteins compared to FSC^Control^-EVs in all analysis results. Error bars represent standard deviation. *, P <0.05; **, P <0.01; ***, P<0.001; ****, P<0.0001; Student's t-test.

The complement system contributes to local inflammation, pathogen elimination and death, and the formation of the subsequent adaptive immune response through three main functions, including opsonization, chemotaxis, and lysis 37. Most of the complement proteins in the serum are produced and secreted by liver cells, and a small amount of them is secreted from endothelial cells, epithelial cells, and immune cells such as monocytes, macrophages, and DCs in a local area where the serum is restricted 38. However, there has been no report on whether stem cells secrete complement proteins. From the results of protein antibody microarray and protein absolute quantification analysis on the components in FSC^Control^-EVs and NAbs-FSC-EVs, we confirmed that various complement proteins related to three activation pathways were induced in NAbs-FSC-EVs at a higher level than FSC^Control^-EVs (**Figure 5A-C**). Our results show that FSCs in early pregnancy, before the innate immune system is fully established, can form the self-defense system to protect themselves from unexperienced antigens by secreting NAbs, including IgG3 and IgM, and various complement proteins.

### Identification of the characteristics as an immune system of the self-defense mechanism formed by NAbs-FSCs

**Figure 6:**
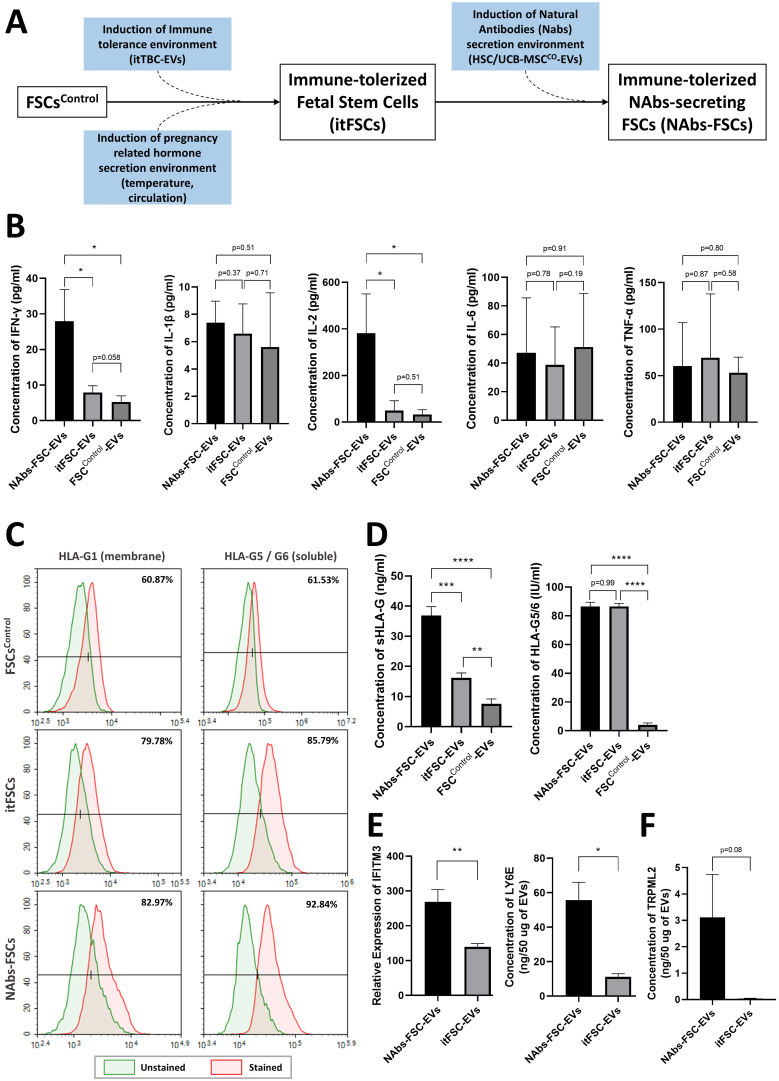
** Comparison of secretion characteristics of cytokines, HLA-G, and anti-viral proteins in FSCs established in various culture conditions. (A)** Various FSCs established in different culture conditions to characterize the self-defense mechanism formed by NAbs-FSCs. **(B)** Cytokine analysis results of EVs from FSCs obtained under each culture condition using human cytokine array. The secretion levels of pro-inflammatory cytokines (IL-1β, IL-6, TNF-α) in FSC^Control^-EVs, itFSC-EVs, and NAbs-FSC-EVs were similar, but the secretion levels of Th1 cytokines (IFN-γ and IL-2), showing increased expression in asymptomatic infected person to COVID-19, were the highest in NAbs-FSC-EVs. **(C)** The results of flow cytometry analysis to compare the expression aspects of membrane-bound HLA-G1 and intracellular soluble HLA-G5/G6 in each FSC using 87G (detecting β2m of HLA-G1 and HLA-G5 isoforms) and 2A12 (detecting intron 4 of soluble HLA-G5 and HLA-G6 isoforms) Abs. Similar to the previous study results 22, all HLA-G expressions were increased in NAbs-FSCs and itFSCs compared to FSCs^Control^. In addition, the expression of HLA-G in NAbs-FSCs was also higher than in itFSCs due to the effect of increased IFN-γ stimulation. **(D)** sHLA-G (shedding HLA-G1 and HLA-G5) and HLA-G5/6 concentration analysis results of EVs from FSCs obtained under each culture condition through ELISA analysis using MEM-G/9 (detecting β2m of HLA-G1 and HLA-G5 isoforms) and 5A6G7 (detecting intron 4 of soluble HLA-G5 and HLA-G6 isoforms) Abs, respectively. Similar to the previous study results 22, all HLA-G concentrations were higher in itFSC-EVs and NAbs-FSC-EVs than in FSC^Control^-EVs. However, unlike HLA-G5/6 concentrations, sHLA-G concentration was higher in NAbs-FSC-EVs than itFSC-EVs. **(E)** A comparison of the concentrations of interferon-induced proteins with anti-viral functions in NAbs-FSC-EVs and itFSC-EVs. IFITM3 protein was expressed at a higher level in NAbs-FSC-EVs than itFSC-EVs using SET100 protein antibody microarray, and LY6E protein was contained at a higher level in NAbs-FSC-EVs than itFSC-EVs according to ESI-Q-TOF MS/MS analysis. **(F)** A comparison of absolute quantification of another anti-viral protein (TRPML2) in NAbs-FSC-EVs and itFSC-EVs using ESI-Q-TOF MS/MS. Error bars represent standard deviation. *, P <0.05; **, P <0.01;***, P<0.001; ****, P<0.0001; Student's t-test.

To identify the characteristics as an immune system of the self-defense mechanism formed by NAbs-FSCs and to investigate the effect of HSC/UCB-MSC^CO^-EVs applied in new *ex-vivo* culture conditions as the replacement of immature fetal immune cells to establish NAbs-FSCs, we performed cytokine array analysis on FSC^Control^-EVs, immune-tolerized FSCs-derived EVs (itFSC-EVs), and NAbs-FSC-EVs secreted from three FSCs established under different culture conditions as shown in **Figure 6A**. As a result, we confirmed that the secretion patterns of pro-inflammatory cytokines such as interleukin (IL)-1β, IL-6, and tumor necrosis factor (TNF)-α were similar in EVs from three FSCs, but Th1 cytokine, IL-2 and interferon (IFN)-γ, were secreted in NAbs-FSC-EVs at a higher level than itFSC-EVs and FSC^Control^-EVs (**Figure 6B**). In these results, we noted that IFN-γ was secreted at a higher level in NAbs-FSC-EVs, obtained from FSCs cultured in new *ex-vivo* culture conditions including HSC/UCB-MSC^CO^-EVs, than in itFSC-EVs, because IFN-γ promotes the transcription of the HLA-G gene together with PR 39, and at the same time, acts as a transcriptional factor for various anti-viral proteins that affect mainly on the cell membrane and inside the cell, unlike Igs, NAbs, and complement proteins which are responsible for the protection outside the cell.

To verify the effect of IFN-γ on HLA-G expression and secretion in FSCs, we compared the expression aspects of HLA-G proteins in each FSC through flow cytometry analysis. As a result, we confirmed that the expressions of membrane-bound HLA-G1 and intracellular soluble HLA-G5/G6 were more increased in NAbs-FSCs than itFSCs (**Figure 6C**). Moreover, to compare the secretion aspects of HLA-G proteins in each FSC, we performed ELISA analysis for the content of HLA-G proteins on each EVs. The results showed that sHLA-G (shedding HLA-G1 and soluble HLA-G5) were secreted more in itFSC-EVs than in FSC^Control^-EVs, and in NAbs-FSC-EVs than in itFSC-EVs, but HLA-G5/G6 were secreted at similarly higher levels in NAbs-FSC-EVs and itFSC-EVs than FSC^Control^-EVs (**Figure 6D**), which can be interpreted as a difference due to the membrane-bound HLA-G1. Since itTBC-EVs, including PR which promotes HLA-G gene transcription, were equally applied in both *ex-vivo* culture conditions to establish itFSCs and NAbs-FSCs, we hypothesized that the difference in shedding HLA-G1 in NAbs-FSC-EVs and itFSC-EVs was due to the effect of different amounts of IFN-γ secreted from each FSC.

To verify this, we performed PAA and protein absolute quantification analysis and compared the contents of IFN-inducing proteins in NAbs-EVs and itFSC-EVs. As a result, we confirmed that the contents of IFN-inducing proteins, including interferon-inducible transmembrane protein 3 (IFITM3) and lymphocyte antigen 6 family member E (LY6E), known to play an anti-viral role in the cellular and endosomal membranes 40, 41, were induced at a higher level in NAbs-FSC-EVs than itFSC-EVs (**Figure 6E**). In addition, we demonstrated that transient receptor potential mucolipin subfamily member 2 (TRPML2), which is expressed only in recycled endosomes similarly to interferon-inducing proteins and is known to inhibit virus replication through activation of anti-viral autophagy 42, was induced at a higher level in NAbs-FSC-EVs than itFSC-EVs (**Figure 6F**).

Our results suggest that a more complex and sophisticated self-defense mechanism in the cell membrane and inside the cell by various anti-viral proteins induced by increased IFN-γ stimulation in addition to the extracellular defense mechanism by secretion of NAbs and complement proteins from NAbs-FSCs exists as an immune system. In addition, our findings that NAbs, complement proteins, and various anti-viral proteins are all contained in immune-tolerized EVs expressing various HLA-G isoforms suggest that they may propose a safe response strategy not causing unnecessary immune rejection to patients in the prevention and treatment of various infectious diseases, as identified through previous study 22.

## Discussion

Although many studies have been made through long-term COVID-19 pandemic, conflicting reports have been published on newborns and prenatal fetuses born from mothers infected with SARS-CoV-2 due to many limitations, such as difficulties in designing large-scale studies and ethical issues. However, data accumulated to date on vertical infections 43, 44 were obtained in the last trimester of pregnancy and after birth. Little study has been done on early pregnancy before the fetal immune system was fully established, especially on Igs that protect the fetus against external pathogens.

As a result of culturing FSCs in the first trimester of pregnancy in new *ex-vivo* culture conditions simulating *in vivo* environment of FSCs co-existing with the TBC layer as a protective barrier against external pathogens from mother and undeveloped fetal immune cells, we confirmed that various Igs, including IgG3, IgG, IgM, and IgA, known to be produced only in mature B cells, are induced in NAbs-FSCs. We demonstrated that the B cell-specific proteins such as OCT-1/2, NF-kb, CstF, and Igll1, which promote the transcription and secretion of Igs, complement proteins playing an essential role in the innate immunity along with NAbs, including IgM and IgG3, and various anti-viral proteins are also induced. From these findings, we verified that FSCs, before the immune system was thoroughly developed, can form a perfect primal immune system to protect themselves from foreign pathogens.

As below, we propose the three protection mechanisms for the new immune system, which we demonstrated, never mentioned in the existing immunity.

**Figure 7:**
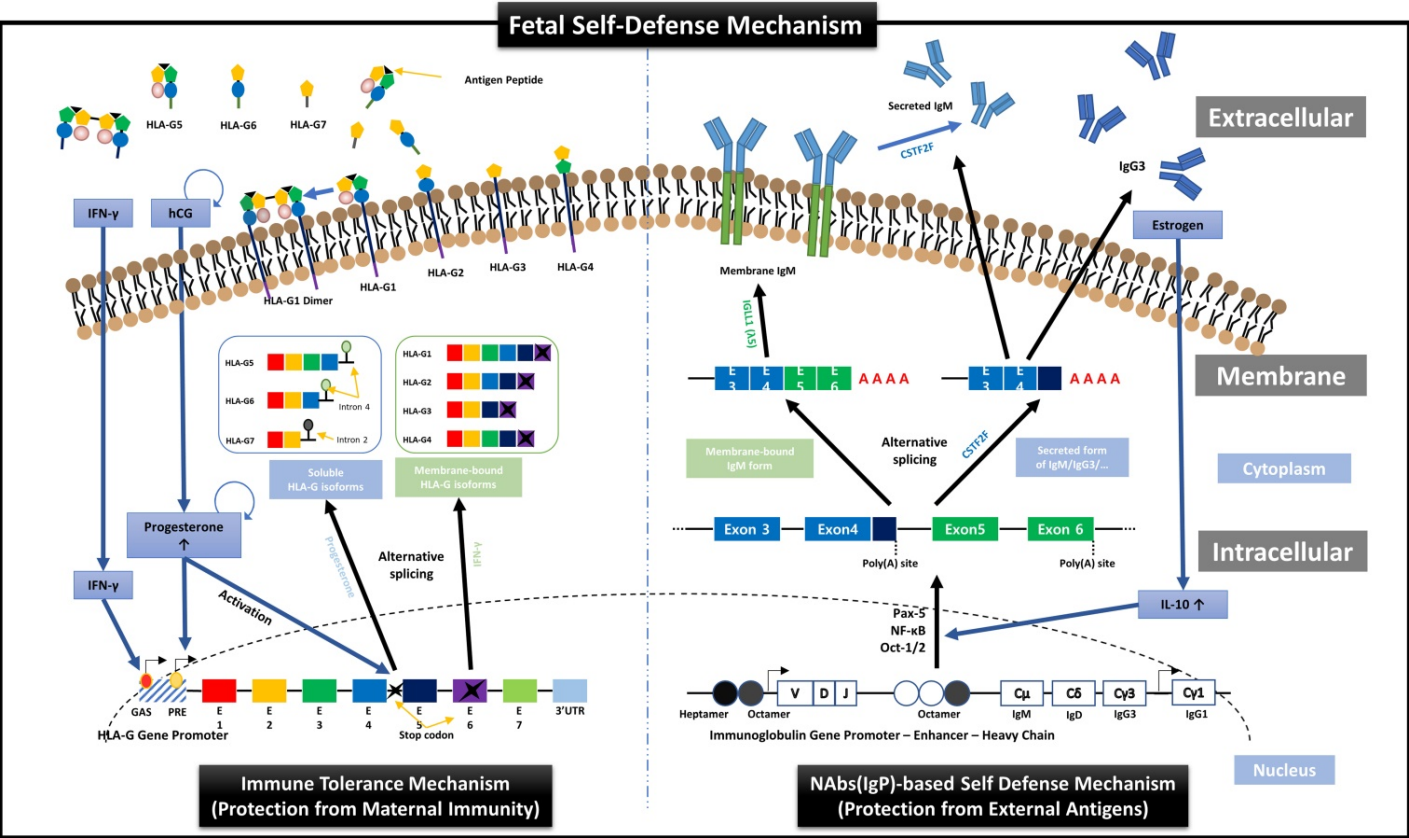
** Overview of HLA-G-based immune tolerance environment and NAbs-based self-defense mechanism induced by hormones secreted during pregnancy.** During pregnancy, the fetus must protect itself from various foreign pathogens and the maternal immune system. Protection from the maternal immune system is achieved by an immune tolerance environment induced by various HLA-G isoforms secreted mainly by EVTs, which continuously infiltrate the maternal decidua. The transcription, alternative splicing, and secretion of soluble HLA-G isoforms, which can induce extensive immune tolerance, are induced by the pregnancy-related hormones hCG and PR 22, and the transcription and expression of membrane-bound HLA-G, which induces local immune tolerance, is also upregulated by IFN stimulation (the left panel). On the other hand, we suggest that before the immune system establishment, the fetal self-defense mechanism against foreign pathogens can be induced by various factors, including IL-10, Pax-5, NF-κB, and Oct-1/2, that promote the transcription of Igs, especially NAbs, such as IgG3 and IgM, which have the most outstanding anti-viral effector functions, through the pathway triggered by ER, another pregnancy-related hormone secreted from TBCs (the right panel). Therefore, our results demonstrate that hormones secreted during pregnancy may induce the different immune systems that fully protect the fetus from maternal immune system and foreign antigens.

First, we suggest the new perspective of fetal protection mechanism by hormones secreted for maintaining normal pregnancy. In other words, just as the expression and secretion of HLA-G proteins that protect the fetus from the maternal immune system are induced by hCG and PR secreted from TBCs 22, we demonstrated the transcription and secretion of Igs that protect the fetus from foreign antigens could be induced by ER. HLA-G is a non-classical human leukocyte antigen G class I protein and responsible for inducting the immune tolerant environment to suppress the immune responses by binding to inhibitory receptors of all immune cells including T, NK, B, and dendritic cells, macrophages, and monocytes, with much higher affinities than other HLA class I molecules. As shown in the left panel of **Figure 7**, HLA-G is affected by IFN-γ and PR during transcription and divided into membrane-bound isoforms (HLA-G1/2/3/4) and soluble isoforms (HLA-G5/6/7), which are responsible for inducing local immune tolerant environment by cell-to-cell contact (direct) and extensive immune tolerant environment by antigen-presenting cells (indirect) in a wider range, respectively, resulting in protecting the fetus from maternal immune system during pregnancy 22. In addition, our results in this study, as shown in the right panel of **Figure 7**, proved for the first time that the mechanism that ER increases the secretion of IL-10, which promotes Igs production in B cells 45, 46, was already applied to FSCs before B cell differentiation.

In addition, we have identified for the first time that EVs secreted from fetal stem cells contain NAbs. The results of flow cytometry analysis for cells and EVs indicate that FSCs have a unique characteristic to secrete EVs containing NAbs, and newly established *ex-vivo* culture conditions highlight this characteristic more clearly. In particular, our results that the concentrations of IgG3 and IgM in NAbs-FSC-EVs gradually increased with proceeding sub-cultivation in newly established *ex-vivo* culture conditions are very similar to the results of our previous study (22), in which the secretion of HLA-G continuously increased as the subculture progressed in TBCs cultured in maternal-fetal interface-like *ex-vivo* culture conditions containing pregnancy-related hormones, hCG and PR promoting HLA-G transcription. Therefore, it can be said that the expression and secretion of NAbs in FSCs are continuously induced by ER, acting as a trigger for transcription of NAbs contained in newly established *ex-vivo* culture conditions. Meanwhile, the reason that the difference in IgM level between both EV groups is lower than that between both FSC groups seems to be due to the limitation of flow cytometry technique for EV analysis that has to analyze IgM levels in the membrane and inside of EVs at once.

**Figure 8:**
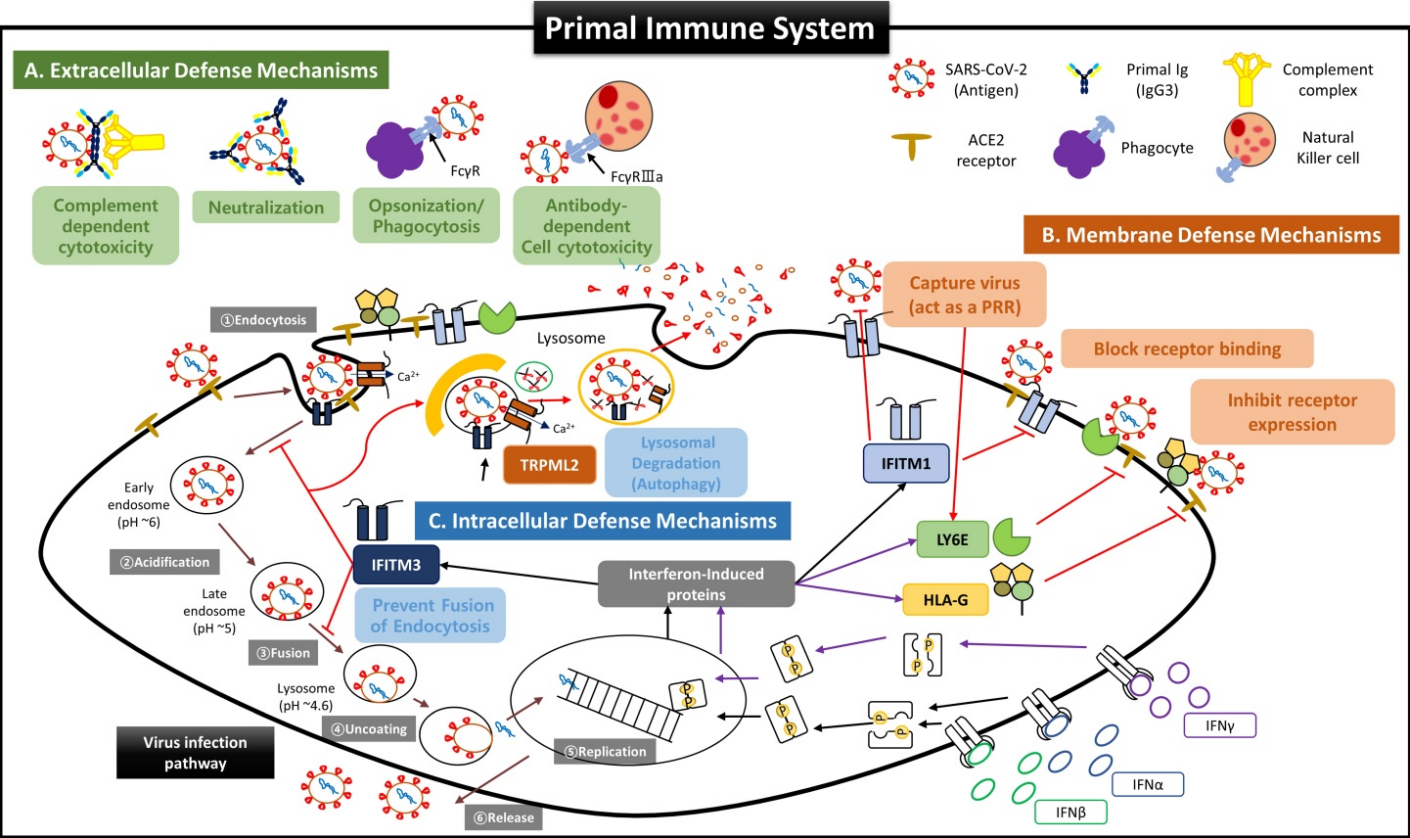
** Suggested Primal Immune System as a Self Defense Mechanism of Fetal Stem Cells before the establishment of innate and adaptive immune systems.** Since virus invasion and infection into host cells are affected by the host's innate and adaptive immune responses, it was recognized that the fetus was very susceptible to viral infection before forming innate and adaptive immune systems. However, our results for FSCs in early pregnancy suggest that the fetus possesses very complicated and sophisticated self-defense mechanisms (primal immune system) at the cellular level exist in addition to the protective function of the placental TBC, which was considered as the only barrier to protect the fetus from external infections so far. **(A)** Extracellular defense mechanism by secretion of NAbs and complement proteins. Our results show that FSCs in early pregnancy can produce and secrete various NAbs, including IgP (fetal IgG3) polyreactive to unexperienced antigens, along with complement proteins to eliminate external pathogens immediately by CDC and ADCC, before umbilical cord generation, the main delivery route of maternal IgG, and before the complete development of B cells. In particular, IgP secreted from early pregnancy FSCs was proved to have the primal Ig repertoire that can instantly recognize various antigenic epitopes pathogens. Furthermore, IgP has the most outstanding Fc effector function to induce ADCC by binding with the highest affinity to Fcγ receptor of NK cells. These functions of IgP may contribute to much effective removal of the infectious agents. Also, we demonstrated that itPG-FSCs could secrete various NAbs such as IgP, IgM, and IgA as they contained factors, including Igll1, Pax5, and Cstf, that promote the secretion of soluble Igs. This finding may indicate that the primal immune system can establish the protective mechanism against various infection routes such as the upper respiratory tract in addition to the placenta and maternal blood. **(B)** The defense mechanism in the cellular and endosomal membranes to inhibit virus entry and replication by IFN-inducing proteins. Over the past decade, several IFN-inducible proteins, including interferon-inducible transmembrane family (IFITMs), ArfGAP with dual pleckstrin homology (PH) domains 2 (ADAP2), gamma-interferon-inducible lysosome/endosome-localized thiolreductase (GILT), and LY6E, have been known to regulate the infectious entry of various viruses 40. Still, few studies have been performed on the fetus during pregnancy. However, we proved through new *ex-vivo* culture conditions containing HSC/UCB-MSC^CO^-EVs that the expressions of IFN-inducible proteins such as IFITM3, LY6E, and HLA-G were increased in fetal stem cells by increased IFN stimulation. Our results that itPG-FSC-EVs contained a higher level of LY6E and IFITM3 than FSC-EVs suggest that FSCs also have a sophisticated self-protection mechanism that suppresses the viral infection by LY6E mainly expressed in the plasma membrane at the beginning of virus entry and by IFITM3 expressed in the endosomal membrane after cell entry. In addition, membrane-bound HLA-G, whose expression is increased by IFN stimulation, may inhibit the relative expression of receptors that can be used as entry pathways of viruses and protect infected host cells by inducing delayed immune response through trogocytosis by contact with immune cells 48. **(C)** Intercellular defense mechanism to inhibit virus transmission by activating anti-viral autophagy. Transient Receptor Potential Mucolipin Subfamily (TRPMLs) are proteins constituting endosome cation channels and perform various physiological functions. TRPML1 is receiving attention as a target molecule that inhibits the fusion of the SARS-CoV-2 envelopes and endosomes 62, and a novel role of TRPML2, only expressed in the recycled endosome, in the innate immune response was recently revealed 42, but there is still little study on fetal stem cells during pregnancy. From our results, the expression of TRPML2 was increased in itPG-FSC EVs. We intend to suggest a new mechanism that the viral antigen information degraded by IFITMs upregulated by interferon stimulation acts as a pattern recognition receptor (PRR), thereby increasing TRPML2 expression 40, 42. In addition, upregulated TRPML2 may contribute to the intracellular protective mechanism that can prevent the replication of infected inhibits virus by binding to the endosomes containing the virus entering the cell, thereby inhibiting the entry of the virus into the cell nucleus and by inducing the degradation through anti-viral autophagy (lysosomal degradation).

Second, we propose that the self-defense mechanism of FSCs is acting as an “immune system”. FSCs in early pregnancy before developing the adaptive immune system by T and B cells should immediately remove the foreign antigens when the infection occurs while minimizing the damage caused by NK cells, which account for most fetal immune cells to maintain normal development. In this regard, we suggest that the following protection mechanisms by various anti-viral factors contained in NAbs-FSC-EVs exist as a sophisticated self-defense system that works organically as below (**Figure 8**);

- Protective functions outside cells by NAbs and complement proteins;
- Protective functions in the cell membrane by regulating the expression of receptors as the co-expressions of angiotensin-converting enzyme 2 (ACE 2) and transmembrane protease serin subtype 2 (TMPRSS2), which are essential for intracellular entry of SARS-CoV-2, are downregulated in placental trophoblasts, resulting in a low infection rate 15, 47;
- Protective functions inside cells that inhibit the replication of infected viruses and induce a delayed immune response by NK cells during the degradation process of activated anti-viral autophagy 48.

Although we couldn't obtain the analysis results for more IFN-inducing proteins in NAbs-FSC-EVs due to the limitation of the protein library in PAA we used, we expect a much more elaborate defense system will protect FSCs from foreign infectious agents than our results. Therefore, we propose to define the defense system of FSCs as a new term, “Primal Immune System”, to distinguish from the existing innate and adaptive immune systems.

Third, the most notable result is that IgG3, one of NAbs, was detected in NAbs-FSC-EVs only obtained under newly established *ex-vivo* culture conditions. NAbs are preimmune Abs generated without antigen stimulation and act as a primary line of defense against infection because they can immediately nonspecifically cross-react with various exogenous antigens 49. IgM is known as a representative NAbs, but our study shows that early pregnancy FSCs can secrete IgM, IgA, IgG, and especially IgG3, which is recently attracting attention as an ideal alternative to existing Ab-based immunotherapies 18. IgG3 has many differences that distinguish it from other IgG subclasses.

- IgG3 has a long hinge region consisting of 11 disulfide bonds to provide extended flexibilities and reaches to bind various antigens of wide ranges;
- IgG3 has various functions, including significant improvement of Fc effector activity, broader neutralizing efficacy, and intensively inducing adaptive immune responses, by high binding affinities to Fcγ receptors of immune cells and complement protein C1q, which induce Ab-dependent cell cytotoxicity (ADCC) and complement-dependent cytotoxicity (CDC) 50-54;
- IgG3 has a more remarkable neutralization ability despite structural modification of endocytic virus into cells due to the property to form aggregates more easily than other IgG subclasses at low pH and maintain binding to receptors 55-57.

However, a more critical point than these structural features of IgG3 is that FSCs in early pregnancy possess the most naive, inexperienced Ig repertoire that can immediately recognize and respond to all foreign antigens. The primary Ig repertoire, which is formed in the early stage of B cells development at about 26 weeks of pregnancy 58, 59, gradually has antigen-specific diversities and becomes increasingly difficult to expand the repertoire for unexperienced new antigens because the number of naive B cells contributing to the formation of the primary Ig repertoire decreases, while antigen-experienced B cells accumulate due to various acquired factors with aging 60. These are supported by many studies showing that children's immune systems respond better to COVID-19 than the old and adults 11-14 and that Ig repertoires responding to COVID-19 exist even in some adults not infected with COVID-19 61.

**Figure 9:**
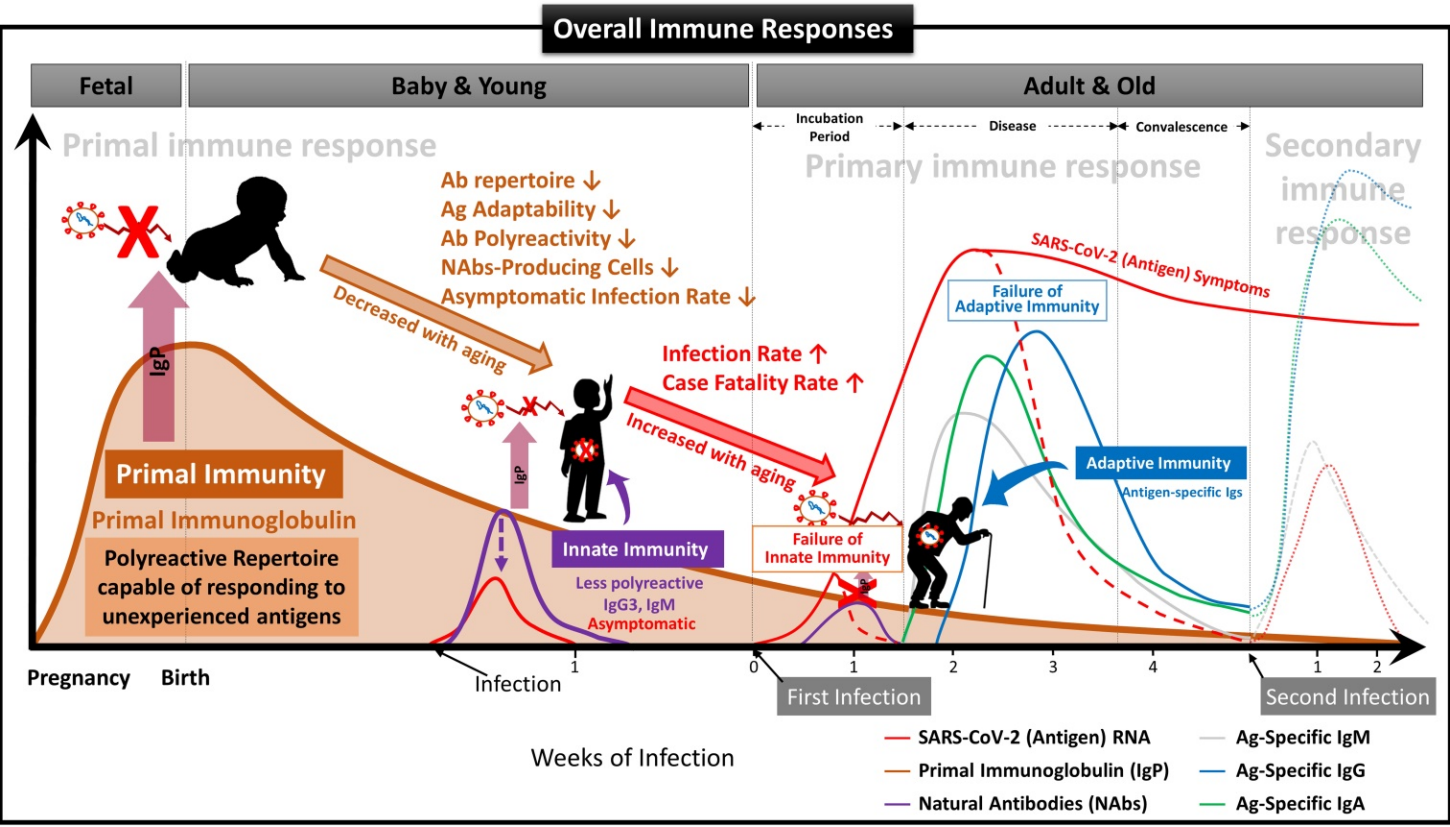
** Suggested Immune System for the whole life.** To date, the antibody-based humoral immune system against foreign pathogen infection is divided into three categories according to the infection stage 21. The first is the immediate innate immune response by NAbs already present in the body before antigen exposure, and it acts as the first line of defense against infectious agents. The second is an extrafollicular "innate-like" antibody response induced within the early few days after antigen exposure, which serves to eliminate the infection temporarily until the specialized antibody response matures. Lastly, it is an acquired antibody response that appears delayed 1-2 weeks after infection by highly assembled antibodies through somatic cell hypermutation and class switch recombination processes. However, we found that the primal immune system by IgP existed before the fetal immune system establishment during pregnancy, and the adaptabilities of innate and adaptive immune systems against foreign antigens depend on the primal repertoire that was experienced and formed during this period. **(A)** Primal Immunity. According to the study for healthy fetuses in early pregnancy 58, circulating B lymphocytes in fetal blood at 12 weeks of gestation have a diverse BCR repertoire, and immunoglobulin heave chain variable region gene (IGHV)-containing clones analyzed at 26 weeks of gestation are similar to those of healthy infants. It was confirmed that it exists in proportion, but this can be called the primary repertoire. On the contrary, the repertoire of NAbs, including IgG3 secreted from FSCs obtained around 10 weeks of pregnancy, which we confirmed through this study, has not experienced any antigens except for themselves, and in other words, can respond to any antigens. Therefore, it can be a primal repertoire, and it will be gradually diversified to the primary repertoires with antigen-specific diversities due to modifying factors such as Abs transmitted from the mother, food and environmental factors, vaccinations, infections, or diseases 58. **(B)** Innate Immunity. The primary repertoire formed from the Primal repertoire in early pregnancy plays a critical role in manifesting symptoms after infection in the later innate and adaptive immune systems. Younger children who have a relatively similar repertoire to the Primal repertoire can react immediately to new antigens and eliminate them before the adaptive immune system acts. However, since birth, the number of naïve B-1 cells that secrete natural antibodies acting on the innate immune system, the ability to secrete antibodies, and the adaptability of the repertoire to new antigens gradually decrease with age. The ability to cope with new viruses, such as SARS-CoV-2, also decreases with age 60. For this reason, many children and younger generations have asymptomatic infections of COVID-19, and even young children are well adapted to the mutant virus 63, while the older, the more symptomatic and severe the proportion of patients increases. **(C)** Adaptive Immunity. The evasion mechanisms to the initial innate immunity of all viruses, including SARS-CoV-2, and the resulting delayed priming of T cell responses and adaptive immunity are receiving attention as the main factors that increase the severity and fatality of COVID-19 with age 64. In other words, as the naïve T cell pool gradually decreases with age, it is difficult to generate a T cell response capable of recognizing a new antigen. This assumption can be supported by the findings that systemic excessive immune pathology, including cytokine storms, is caused by an explosive innate immune response activated to replace the delayed adaptive immune system 6. In particular, the delayed adaptive immune response can cause taste and olfactory abnormalities in asymptomatic and mild patients accounting for more than half of SARS-CoV-2 infections 65, so the most effective prevention and treatment for various infections including SARS-CoV-2 It can be said that the strategy lies in an effective innate immune system capable of triggering a normal adaptive immune response. From this point of view, it is expected that our research results, which first proposed the concept of the primal immune system before the formation of the innate immune system, will contribute to finding a fundamental solution to protect humanity against unknown infectious agents in the future. Another important finding in our results is that IgA is also contained in itPG-FSC-EVs secreted from FSCs in early pregnancy. It can be expected to provide an expanded variety of applicability to various infection pathways, such as the upper respiratory tract in the existing IgG-centered Ab therapeutics.

We have demonstrated that NAbs-FSCs, established in this study by culturing FSCs obtained at around 10 weeks of gestation, that is, the stem cells in the early stage of HSCs that can differentiate into B cells, could secrete a variety of NAbs, including IgG3. Since these NAbs are not almost exposed to any antigens, they may have the "Primal Ig Repertoire" that can form a full range of primary Ig repertoire that expands later in the B cell development stage against all antigens. Therefore, to differentiate IgG3, a NAb secreted from FSCs in the early stages of pregnancy and having a primal repertoire, from the existing IgG3, which is known to be secreted from B-1 cells, we suggest that it should be redefined with a new classification and terminology as “Primal Immunoglobulin (IgP)”. In addition, we would like to define IgP-secreting FSCs established under new *ex-vivo* culture conditions in this study as “immune-tolerized primal immunoglobulin secreting FSCs (itPG-FSCs)”. Also, considering the characteristics of the primal immune system by the newly defined IgP, we propose a human immune response system against external infections, including SARS-CoV-2, as shown in **Figure 9**.

Lastly, through additional studies and clinical trials of “itPG-FSC-EVs” not causing immune rejection, as demonstrated in the previous study 22, and containing all of the various anti-viral factors constituting the newly proposed “Primal Immune System”, we expect that our results become new immunological strategies that can be applied to fundamentally preventive protection and treatment against foreign antigens of various infection pathways, including SARS-CoV-2.

## Figures and Tables

**Table 1:** Comparative Analysis of IgG3 and IgM in culture medium and EVs from various cells

Cell Lines	Concentration of IgG3 (ng/ml)	Concentration of IgM (ng/ml)
FSCs (2D)	< Min	< Min
FSCs (Ex-vivo)	0.5512 ± 0.0290	1.497 ± 0.417
UCB-MSCs (2D)	< Min	< Min
UCB-MSCs (Ex-vivo)	< Min	< Min
^1)^ADSCs (2D)	< Min	< Min
ADSCs (Ex-vivo)	< Min	< Min
^2)^BM-MSCs (2D)	< Min	< Min
BM-MSCs (Ex-vivo)	< Min	< Min
^3)^UC-MSCs (2D)	< Min	< Min
UC-MSCs (Ex-vivo)	< Min	< Min
HSCs (2D)	< Min	< Min
HSC/UCB-MSC^CO^-EVs (Co culture)	< Min	< Min
AF/AM-MSC^CO^-EVs (Co culture)	< Min	< Min
itTBC-EVs	< Min	< Min

1) ADSC, Adipose-derived stem cells; 2) BM-MSC, Bone marrow derived mesenchymal stem cells; 3) UC-MSC, umbilical cord-derived mesenchymal stem cells.

## Abbreviations

Ab: antibody
ADCC: Ab-dependent cell cytotoxicity
AF/AM-MSC^CO^-EV: extracellular vesicle from serum-free co-cultivation of human amniotic fluid and amniotic membrane derived mesenchymal stem cells
BCR: B cell receptor
CDC: complement-dependent cytotoxicity
CFR: case fatality rate
COVID-19: coronavirus disease 2019
CSTF: cleavage stimulation factor
DC: dendritic cell
ELISA: enzyme-linked immunosorbent assay
ER: estrogen
ESC: embryonic stem cell
EV: extracellular vesicle
EVT: extravillous trophoblast
FBS: fetal bovine serum
FSC: fetal stem cell
FSCs^Control^: FSCs cultured in general 2D-plate
FSCs^Control^-EV: EV secreted from FSCs^Control^
FSCs^Ex-vivo^: FSCs cultured in new *ex-vivo* culture conditions
HA: hyaluronic acid
hAF-MSC: human amniotic fluid-derived mesenchymal stem cell
hAM-MSC: human amniotic membrane-derived mesenchymal stem cell
hCG: human chorionic gonadotropin
HLA-G: human leukocyte antigen G
HSC: human hematopoietic stem cell
HSC/UCB-MSC^CO^-EV: EV from serum-free co-cultivation of HSCs and human umbilical cord blood mesenchymal stem cells
IFITM3: interferon-inducible transmembrane family member 3
IFN: interferon
Ig: immunoglobulin
IGLL1: immunoglobulin lambda-like polypeptide 1
IgP: primal Ig
IL: interleukin
itFSC: immune-tolerized FSC
itFSC-EV: EV secreted from itFSCs
itPG-FSC: immune-tolerized IgP-producing FSC
itPG-FSC-EV: EV containing IgP secreted from itPG-FSCs
itTBC: immune-tolerized trophoblast cells
itTBC-EV: EV secreted from itTBCs
LY6E: lymphocyte antigen 6 family member E
MSC: mesenchymal stem cell
NAb: Natural antibody
NAbs-FSC: FSC secreting NAbs
NAbs-FSC-EV: EV secreted from NAbs-FSCs
NF-κB: nuclear factor kappa-light-chain-enhancer of activated B cells
NK: natural killer
NTA: nanoparticle tracking analysis
OCT: octamer transcription factor
PAA: protein antibody array
Pax5: paired box protein 5
PBMC: peripheral blood mononuclear cell
PBS: phosphate buffered saline
PR: progesterone
SARS-CoV-2: Severe acute respiratory syndrome coronavirus 2
SEM: scanning electron microscope
sHLA-G: shedding HLA-G1 and HLA-G5
STB: syncytiotrophoblast
TBC: trophoblast cell
TEM: transmission electron microscope
TRPML2: transient receptor potential cation channel mucolipin subfamily member 2
UCB-MSC: human umbilical cord blood MSC
